# Correction to “In silico validation revealed the role of *SCN5A* mutations and their genotype–phenotype correlations in Brugada syndrome”

**DOI:** 10.1002/mgg3.2487

**Published:** 2024-07-12

Pham, H. M., Dang, D. P., Ta, T. D., Le, T. P., Phan, D. P., Trinh, H. A., Tran, T. V., Luong, T. L. A., Nguyen, H. M., Bui, T.‐H., Tran, T. H., Ta, T. V., & Tran, V.‐K. (2023). In silico validation revealed the role of *SCN5A* mutations and their genotype–phenotype correlations in Brugada syndrome. *Molecular Genetics & Genomic Medicine*, 11, e2263. https://doi.org/10.1002/mgg3.2263

In the originally published article, authors Duy Phuong Dang and Dinh Phong Phan were erroneously identified as Duy Phuong Nguyen and Phong Hai Phan, respectively. The online version of this article has been updated.

We apologize for this error.

